# Sexual Dysfunction in Individuals with Early-Onset Parkinson’s Disease in Ethiopia: Gender Differences and Correlation with Anti-Parkinson’s Medication, Stigma, and Distress

**DOI:** 10.3390/healthcare14020153

**Published:** 2026-01-07

**Authors:** Arefayne Alenko, Morankar Sudhakar, Legese Chelkeba, Ines Keygnaert

**Affiliations:** 1 International Center for Reproductive Health, Department of Public Health and Primary Care, Ghent University, 9000 Ghent, Belgium; ines.keygnaert@ugent.be; 2 Department of Health Behavior and Society, Jimma University, Jimma P.O. Box 378, Ethiopia; morankarsn@yahoo.com; 3 Department of Pharmacology and Clinical Pharmacy, College of health sciences, Addis Ababa University, Addis Ababa P.O. Box 3434, Ethiopia; legese.chelkeba@gmail.com

**Keywords:** sexual dysfunction, depression, stigma, Parkinson’s disease, sub-Saharan Africa

## Abstract

**Background**: Sexual dysfunction (SD) affects up to two-thirds of individuals with early-onset Parkinson’s disease (PD), yet it remains underexplored in developing countries where stigma, depression, and treatment side effects may worsen its burden. This study investigated the magnitude and correlation of SD in early-onset PD. **Methods**: A cross-sectional study included 74 individuals with PD onset at ≤55 years of age. SD was assessed using the Medical Outcomes Study Sexual Functioning Scale, alongside interviews on sexual experiences after initiating PD medication. Prevalence was estimated descriptively, and Spearman’s correlation identified correlates of SD. **Results**: Half of participants reported SD, including lack of sexual interest (52.7%), difficulty relaxing or enjoying sex (52.7%), and impaired arousal (50%). Among men, 48% experienced erectile problems, while 44% of women reported difficulty achieving orgasm. After starting anti-Parkinson’s medication, 16% noted markedly reduced sexual desire, whereas nearly 10% reported increased desire. In men, SD correlated with levodopa dose (r = 0.411, *p* < 0.01). In women, SD correlated with stigma (r = 0.389, *p* < 0.05), depression (r = 0.529, *p* < 0.01), and anxiety (r = 0.629, *p* < 0.01). **Conclusions**: One in two individuals with early-onset PD experiences SD, independent of gender. Findings highlight the need for routine sexual health assessment and careful monitoring of treatment side effects. Interventions targeting stigma, depression, and anxiety are critical to improve sexual well-being in this population.

## 1. Introduction

Parkinson’s disease (PD) is the most prevalent neurologic disorder characterized by tremors, rigidity and slowed movement. It is increasingly recognized that non-motor symptoms of PD, mainly sexual dysfunction (SD), are common and can adversely affect intimate relationships, yet they often are not diagnosed nor treated [1]. SD in PD patients can be reduced sexual desire, problems of inability to have sex, and hypersexuality (HS). Hypersexuality is a known form of SD in PD patients and is secondary to dopaminergic medications. Up to 33% PD patients develop HS [2] and up to 52% PD patients reported SD [3]. In PD patients, 98% of HS is due to dopaminergic drugs [4].

The magnitude of SD is higher among male PD patients and those with long duration of illness [3]. However, motor symptoms of PD affect sexual functioning and independence more in females than in males [5]. Erectile dysfunction (ED) and HS are common in men while orgasm dysfunction and reduced libido were common in women [3]. However, it had been concluded that SD is an underreported problem in people with PD [3].

SD in PD patients can vary with age at onset, gender, illness severity and anti-Parkinson’s medication [6,7,8]. The impact of PD on sexual health is enormous when its onset is early in life [7]. In people with early-onset PD, 66% experience lack of sexual desire, and 43% of men experience erectile dysfunction [9]. In addition, HS secondary to levodopa, the mainstay of PD treatment, is another form of SD in male and young-onset PD [8].

Sexual functioning in people with PD has received poor attention so far [10,11]. This is attributable to the severity and progressive nature of PD, which prompts caregivers and researchers to prioritize motor symptoms that influence treatment outcomes and survival, including abnormal body movements and imbalance. Also, individuals with PD often express the overruling feelings related to everyday survival issues, rather than talking about sexuality and intimacy [12]. Sexual activity is an essential part of daily life and intimate relationships, yet it can be greatly influenced by the disease [13].

PD causes psychological distress, such as low self-esteem, changes in body image, and depression, which can directly affect sexuality [14]. The sexual practices were also determined by the emotional impact of the chronicity of the illness than by physical symptoms alone. The majority of patients lost confidence, depressed, and anxious as a result of poor sexual functioning [14].

The time of onset of problems related to sexuality and intimacy among individuals with PD varies with gender, age, and presence of depression. In men, frequency of intercourse, sexual arousal, and sexual satisfaction more commonly deteriorated than in women [15]. Women frequently experience difficulties achieving orgasm and a lack of sexual desire [16]. As a result, they worry about not meeting their partner’s sexual expectations. Avoiding sexual activities can lead to withdrawal from the relationship, increased thoughts of divorce, and dissatisfaction with their sexuality and relationship. Additionally, a longer illness duration may increase moments of tenderness with their partner [16].

Stigma related to sexuality among individuals with chronic illnesses such as PD can lead to distortion of one’s overall perception of sexual functioning and wellbeing. This can be explained in terms of societal misperception and attitude towards disability and sexuality. In this context, persons with disabilities are commonly considered as asexual [17], and unfit sexual/marriage partners [18]. Due to disability, women are being excluded from a life of active sexuality and denial of chances for motherhood [17,18]. Therefore, sexuality and reproductive concerns and aspirations in individuals with the disease are ignored in society, given less attention in health care services and research in sub-Saharan countries.

**Figure 1:**
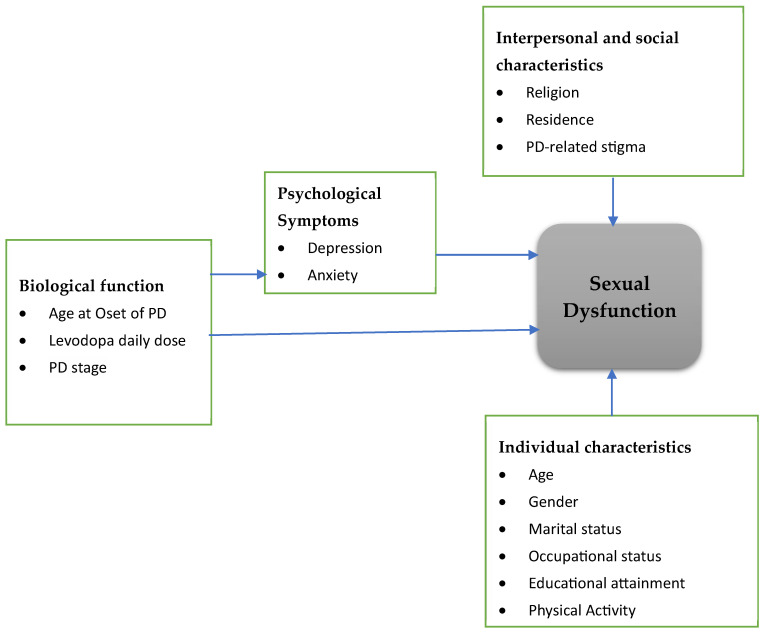
Conceptual Framework.

In sub-Saharan Africa, SD among individuals with PD has been inadequately studied. To our knowledge, no previous study has examined SD in early-onset PD within sub-Saharan Africa. Sexual functioning, particularly in the context of PD-related stigma, anti-Parkinsonian medications, and mental distress, remains under-investigated. Therefore, this study aimed to assess the magnitude of SD, examine the influence of anti-Parkinsonian medications on sexual functioning, and identify the clinical and psychosocial correlates of SD among individuals living with early-onset PD in Ethiopia. The findings are intended to inform the development of targeted interventions to enhance the sexual health and health-related quality of life of people living with PD. A conceptual framework was developed to explain the hypothesized relationships between sexual dysfunction and biological factors (age at PD onset, daily levodopa dose, and PD stage), psychological symptoms (depression and stigma), individual characteristics such as physical activity, and interpersonal or social factors including PD-related stigma (Figure 1).

## 2. Method and Materials

When planning the study and presenting the results, we adhered to the STROBE guideline (Strengthening the Reporting of Observational Studies in Epidemiology) to the transparency and reproducibility of the findings [19]. This study is part of a broader research project titled, “Mental, sexual, and reproductive health consequences of Parkinson’s disease and their impact on quality of life”. A portion of this research project has been published elsewhere [20]. Therefore, there are some methodological similarities, such as the study setting, design, study population, and measurement instruments, across the manuscripts derived from the same project. However, each manuscript addresses distinct research questions and analytical objectives. The current manuscript specifically focuses on sexual dysfunction in people with early-onset PD, whereas the previous papers examined mental health and quality of life across all age groups of PD onset.

### 2.1. Study Setting and Design

The study was carried out in the outpatient departments of Yekatit 12 Medical College, Zewditu Memorial Hospital, St. Hospital Millennium Medical College, and Tikur Anbessa Specialized Hospital. These are tertiary-level hospitals in Addis Ababa, the capital city, where patients with neurological disorders are referred to. We conducted an initial evaluation at various hospitals to determine the number of PD patients receiving follow-up care, the presence of a distinct neurology clinic, and the formal and informal communication channels considered for PD treatment referrals. After examining their medical histories to verify diagnosis and ongoing therapy, we also added PD patients from Parkinson’s patient support organization of Ethiopia (PPSO-E). For those with Parkinson’s disease, PPSO-E offers medical and financial assistance. The study was conducted using a cross-sectional design between 15 July 2023, and 15 March 2024.

### 2.2. Population and Recruitment

**Figure 2:**
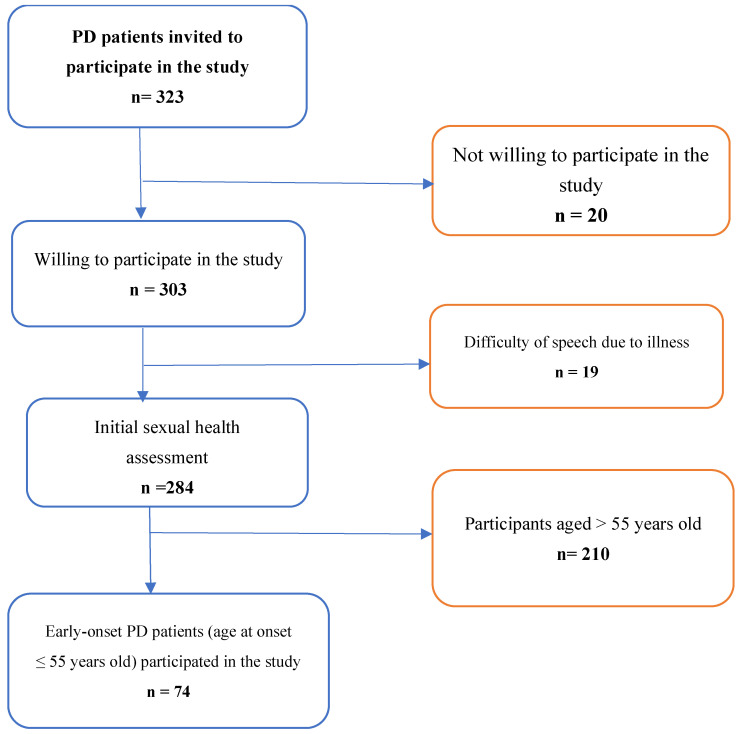
Recruitment of study participants.

According to the ministry of health of Ethiopia report, there were 3402 cases of PD, and 1571 cases of Parkinsonism reported as of mid-November 2023. In this study, all patients attending outpatient treatment at four selected hospitals of Ethiopia and patients engaged in patients’ organization are eligible to participate. Patients with confirmed diagnosis of PD, after reviewing their medical record or chart, were included in the study. However, patients with diagnosis not confirmed were excluded. In addition, we did not include Parkinson’s disease secondary to medication side-effect. Due to lack of clarity in patient registration and follow-up visits, we applied consecutive sampling techniques to recruit participants in selected hospitals. Recruitment procedure of the study participants is shown in Figure 2.

### 2.3. Data Collection Methods and Measurements

We conducted face-to-face interviews using a structured questionnaire containing standard and validated scales. We recruited two clinical nurses for data collection and one health professional with MSc level training to supervise data collection in each selected hospital. We used the KoboCollect v2023.2.4 mobile application for data collection. (https://support.kobotoolbox.org/kobocollect_on_android_latest.html (accessed on 5 December 2023)) The questionnaire was drafted in English and subsequently translated to the local language, Amharic, to collect data. Before data collection, 3 days training and orientation were provided for data collectors and supervisors. Additionally, a back-and-forth translation process was carried out to ensure consistency. We also sought feedback from mental and sexual health professionals to enhance the clarity and cultural appropriateness of the questionnaire. Following the training of data collectors and supervisors, we conducted a pre-test of the Amharic questionnaire to evaluate clarity, cultural appropriateness, and comprehension of the translated items. The pre-test involved a small number of individuals living with PD. Participants were asked to complete the questionnaire and provide feedback on wording, cultural acceptance, and any phrases that felt ambiguous or inconsistent with everyday Amharic usage. Data collectors also documented any difficulties observed during administration. After pretest, minor revisions were made to improve linguistic precision and enhance cultural acceptability. These adjustments included refining specific terms to better reflect locally understood expressions related to sexual functioning and ensuring that phrases were contextually appropriate. No major changes were required, and the refined version was then used for the main data collection.

To avoid repeated interviews, participants’ unique code and name of hospital were posted in the Telegram (Telegram for Andv10, FZ-LLC, Dubai, United Arab Emirates) group immediately after recruitment for interview. Variables and their measurements are listed below.

Sexual Dysfunction: medical outcomes study sexual functioning scale (MOS-SFS) was utilized to determine the magnitude of sexual dysfunction [21]. The MOS-SFS is a four-item Likert scale (1 = “not a problem,” 2 = “little of a problem,” 3 = “somewhat of a problem,” and 4 = “very much a problem”). The items assess lack of sexual interest, inability to relax and enjoy sex, difficulty in becoming sexually aroused, difficulty obtaining or maintaining an erection (men only), and difficulty achieving orgasm (women only). A total score is transformed to 100 and a higher score indicates worse sexual functioning or impairment [21]. Participants who responded 3 (somewhat of a problem) and above in each item were considered as having sexual dysfunction in each domain. MOS-SFS is a valid tool used in different studies to assess sexual functioning [3,21,22]. Although the Amharic version of the scale has not been formally validated, mental and sexual health professionals provided positive feedback regarding its clarity and cultural appropriateness. An internal consistency (Cronbach’s alpha) of MOS-SFS in this study.is 0.83. We assessed the magnitude of sexual dysfunction after starting PD medication, specifically levodopa, by asking: ‘How is your sexual desire after starting PD medication?’ (1 = significantly reduced, 2 = somewhat reduced, 3 = the same/no difference, 4 = somewhat increased, 5 = significantly increased).

Depression and anxiety: Hospital Anxiety and Depression Scale (HADS) was used to assess depression and generalized anxiety [23,24,25]. HADS is a 14-item tool commonly used to screen symptoms of anxiety and depression among patients on follow-up treatment. The 14-item scale has 2 subscales of anxiety (HAD-A) and depression (HAD-D), each containing 7 items. HADS is validated in Ethiopia with reported internal consistency of 0.78 for HAD-A, 0.76 for HAD-D, and 0.87 for the full scale. The higher score, in both cases, indicates the severity of anxiety and depression [23,24,25]. The Amharic version of the Hospital Anxiety and Depression Scale (HADS) has been validated in Ethiopia [23,26].

Stigma: is measured by using the stigma scale for chronic illness 8-item version (SSCI-8) [27]. SSCI is validated across different neurological disorders including PD. It is a linear Likert scale that ranges 1 (never) to 5 (always), with a total score range from 8 to 40 [27]. SSCI-8’s higher score indicates a higher level of stigma. It has been validated as a unidimensional measure of stigma [28]. An internal consistency (Cronbach’s alpha) of SSCI-8 in this study.is 0.90.

PD stage: the staging of PD was classified according to modified Hoehn and Yahr Scale (HYS). According to HYS, PD stage is categorized into stages 1, 1.5, 2, 2.5, 3, 4 and 5 [29].

Moderate intensity physical activity (MIPA): MIPA is measured according to the World Health Organization (WHO) physical activity recommendation for people living with chronic illness [30]. So, participants moderate intensity activities, such as walking very brisk and running, were assessed by multiplying number of days in a typical week and duration of activity in minutes in a typical day [30].

### 2.4. Data Processing and Analysis

We downloaded csv data type from KoboCollect and imported it to IBM SPSS Statistics version 20.0 (IBM Corp., Armonk, NY, USA) for analysis. We used descriptive techniques of analysis to estimate magnitude of different types of sexual dysfunction. Before analysis, data visualization and assumption of normality was checked. After observing a violation of assumption, we decided to run a Spearman’s rank correlation to identify correlates of sexual dysfunction in both genders. Given the non-normal distribution of the data, Spearman’s rank correlation coefficients (correlation cofficient (r) and cofficient of determination (R^2^)) were employed to examine bivariate associations among study variables. The correlation analyses were conducted to explore the direction and strength of associations, rather than to identify independent predictors. Accordingly, no multivariable regression analyses were performed, and the findings should be interpreted as exploratory. Statistical significance was set at *p* < 0.05. The results are presented by using tables, charts, and figures.

### 2.5. Ethical Considerations

This study was conducted in line with the principles of the Declaration of Helsinki. Ethical approval for the study was obtained from the Research and Ethics Review Committee of the Jimma University Institute of Health (Ref. No: JUIH/297/23). All hospitals and patient organizations were formally contacted and granted permission to conduct the study. Both oral and written informed consent were obtained from study participants. For patients who were unable to sign due to hand tremors, only oral consent was collected. Confidentiality and privacy were strictly maintained at all stages of the study, including ensuring privacy in the interview setting or room.

## 3. Results

### 3.1. Socio-Demographic Characteristics Study Participants

A total of 74 PD patients participated in the survey. The mean age of participants with a standard deviation (StD) was found to be 47.84 ± 6.03 years with a minimum age of 34 years and maximum age of 55 years. Most participants were male, 42 (56.8%) whereas more than one-third were married, 29 (39.2%). Twenty-six (35.1%) participants attended primary education only. The majority were Orthodox Christians by their religion, 50 (67.6%) and living in an urban area, 66 (89.2%). Details of their socio-demographic characteristics are presented in Table 1.

### 3.2. Clinical and Psychosocial Characteristics of Study Participants

The mean age at onset of PD (AO) with StD is 42.66 ± 6.27 with a minimum and maximum of 26 and 52 years, respectively. Mean daily levodopa equivalent dose with StD was 591.22 ± 333.16 with a minimum of 0 mg and a maximum of 1500 mg per day. About one-third of participants were at the second stage of PD, 25 (33.8%). Details of clinical and psychosocial characteristics of the study participants are presented in Table 2.

### 3.3. Prevalence of Sexual Dysfunction

**Figure 3:**
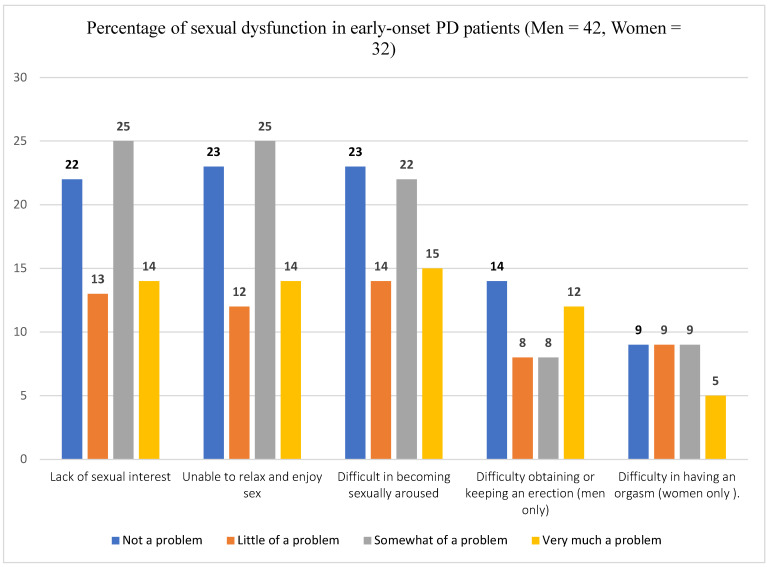
Prevalence of sexual dysfunction in an early-onset Parkinson’s Disease patients.

The prevalence of lack of sexual interest is 52.7% with 95% CI of 40.4–62.2%. Similarly, 52.7% (95% CI: 41.8–63.3) of study participants reported inability to relax or enjoy sex. Half of the participants (95% CI: 39.2–59.6%) reported difficulty of sexual arousal after onset of the disease. Among male participants, 47.6% reported experience of difficulty of erection whereas 43.8% of female participants reported difficulty of orgasm after the onset of the disease. Concerning participants experience of change in their sexual functioning after starting anti-Parkinson’s medication (Sinemet, levodopa-carbidopa), 12 patients (16.2%) reported “significantly reduced sexual desire”, 15 (20.3%) reported “somewhat reduced sexual desire”, and 7 (9.5%) reported “somewhat increased sexual desire” after starting the medication. Prevalence of different types of sexual dysfunction is shown in Figure 3.

### 3.4. Correlates of Sexual Dysfunction

Sexual dysfunction is significantly correlated with a levodopa equivalent daily dose in male participants. There was a moderate positive correlation between daily levodopa dose and sexual functioning among early-onset male PD patients (r = 0.411, *p* < 0.01). The coefficient of determination (R^2^ = 0.169) indicates that daily levodopa dose accounts for approximately 17% of the variance in sexual functioning, suggesting that medication dose is a contributing factor, while the majority of variability is explained by other factors. We found no significant correlation between sexual dysfunction and other factors in male PD patients (Table 3).

In female participants, sexual dysfunction is significantly and positively correlated with experience of PD-related stigma (r= 0.389, *p* < 0.05), depression (r = 0.529, *p* < 0.01), and generalized anxiety (r = 0.629, *p* < 0.01). Therefore, 15%, 28%, and 40% variation in sexual functioning can be explained by the experience of stigma, depression and generalized anxiety, respectively, among early-onset female PD patients (Table 4).

## 4. Discussion

We found that half of the study participants experienced at least one dimension of sexual dysfunction according to the MOS-SF scale: lack of sexual interest, inability to relax or enjoy sex, difficulty of sexual arousal, difficulty of erection in men, and difficulty of orgasm in women. This is the first study to investigate magnitude of sexual dysfunction in Ethiopia as well as Africa more broadly among individuals living with an early onset of PD. In comparison to the current study, both lower and higher prevalence of SD have been reported in developed countries. Among Spanish cohorts with an early-onset PD, SD was present in 70.2% of patients, and sexual dissatisfaction in 65% [31]. Our findings are lower than the report of study conducted in Spain. This might be because of cultural sensitivity of revealing or disclosing sexual functioning in the Ethiopian community. In addition, some methodological differences such as a higher number of study participants (n = 105), and use of self-reported questionnaires (allowing participants to freely report their sexual health status) might contribute to the observed difference. A study conducted in Turkey reported 59% with sexual dissatisfaction in an early-onset PD (age at onset of less than 55 years). This finding is consistent with our estimates (95%CI: 40.4–62.2%). However, compared to the current study, lower prevalence of specific dimensions of sexual dysfunction has been reported in the Turkish study. A study conducted in Turkey reported 23% with problems of sexual desire, 14% with problems of sexual stimulation, 32% with problems of erection or lubrication, and 32% with problems of orgasm [32]. The difference might be due to low sample size (n = 22) and exclusion of patients with depression and anxiety in the Turkish study.

A study conducted in the US among 37 early-onset PD patients (AO ≤ 55 years) showed 37% with clinically significant sexual dissatisfaction. In this US study, the prevalence of difficulty in reaching an orgasm was 30% and erectile dysfunction was 28% [12]. Compared to our study, a lower prevalence of sexual dysfunction was reported in the US study. Methodological differences such as a limited sample size (n = 57), and socio-economic differences such as healthcare coverage and better quality of life may be a reason for the lower prevalence of sexual dissatisfaction. Owing to low socio-economic status, disease illiteracy and poor health care coverage, it is reasonable to see higher prevalence of SD in people with early-onset PD in Ethiopia. Due to lack of research in African early-onset PD patients and SD, this study provides evidence for further research and clinical practice.

We found that anti-Parkinson’s medication (Sinemet, levodopa-carbidopa) had an impact on sexual functioning of the participants. Anti-Parkinson’s medication, particularly levodopa, can reduce sexual desire, if it causes side effects such as fatigue, depression, or anxiety [33]. HS, increased sexual desire or heightened level of sexual interest, is a common form of SD in PD patients secondary to dopaminergic effect of the drug [8,34]. A systematic review showed that the prevalence of HS secondary to anti-Parkinson’s dopaminergic medications ranges from 2% to 7.2% among patients with PD [8]. Our findings are consistent with those of a previous systematic review. However, a higher prevalence has been reported in patients with early-onset PD, with up to 38.3% experiencing HS [34]. Even though there is lack of evidence concerning the extent of impact of anti-Parkinson’s medications on sexual functioning in African countries, with less monitoring of side effects, this study provides an evidence base for further inquiry.

We found a significant correlation between SD and levodopa equivalent daily dose in male participants. Thus, worsening of sexual functioning increases linearly with increment of the levodopa equivalent dose. However, we found no significant correlation between other clinical and sociodemographic variables in male participants. Early-onset PD patients are prone to medication-related complications compared to late-onset PD patients [35]. Studies show levodopa induced hypersexuality in PD patients [8,34]. However, there is no evidence concerning levodopa-induced hyposexuality. Physiological changes such as nausea, dizziness and insomnia, and emotional changes such as depression secondary to levodopa can decrease sexual functioning. The observed association may be associated with other factors such as severity of illness. Therefore, we strongly recommend further study that compares hyposexuality and levodopa dose.

In female participants, sexual dysfunction is significantly and positively correlated with experience of PD-related stigma (r = 0.389, *p* < 0.05). Thus, the 15% variation in sexual functioning can be explained by the experience of stigma in an early-onset female PD patient. It has been reported that early-onset PD patients more frequently experienced perceived stigma than older-onset patients [35]. Stigma related to sexuality among individuals with disabilities can distort their overall sexual self-concept. This stems from societal misconceptions and negative attitudes toward both disability and sexuality. People with disabilities are often wrongly perceived as asexual [17], incapable of reproduction, and unsuitable as sexual or marital partners. Women with disabilities may be excluded from experiencing womanhood, partnerships, active sexuality, and opportunities for motherhood. [17,18]. Similar experience has been reported by Ethiopian mothers with physical disability [36].

In addition to stigma, sexual dysfunction is significantly and positively correlated with experience of depression (r = 0.529, *p* < 0.01) and generalized anxiety (r = 0.629, *p* < 0.01). Thus, the 28% and 40% variation in sexual functioning can be explained by depression and generalized anxiety, respectively. A previous study, ref. [7], also reported that “sexual dysfunction was positively correlated with symptoms of depression and anxiety”. Another study conducted by Kummer A, Cardoso F, and Teixeira AL in 2009 also reported depression as a main predictor of loss of libido in PD patients [9]. By considering specific dimensions of sexual dysfunction, one study reported positive correlation between depression and orgasm dissatisfaction, and anxiety and difficulty of stimulation and orgasm [37]. Beyond its correlation with sexual dissatisfaction, depression is significantly correlated with relationship dissatisfaction in early-onset PD patients [12]. Sexual health is an unmet need in women living with PD [38]. Thus, focusing on both clinical (monitoring drug side effects) and psychosocial aspects (depression, anxiety and stigma) of sexual dysfunction in early-onset PD patients is crucial to provide need-based sexual and reproductive health service.

### Strengths and Limitations

We applied standard and cross-culturally validated tools to measure sexual dysfunction and covariates. In addition, recruiting participants from different institutions, and selecting and confirming primary diagnosis of PD are strengths of the study. However, a rather small sample size, and not including PD patients in the community who have not yet started formal treatment are limitations of the study. Thus, the stability of the correlation estimates may be influenced by the relatively modest sample size. Despite limitations, the findings clearly outline implications for practice and further research in sexual health of early-onset PD patients.

## 5. Conclusions and Practical Implications

One in two early-onset Parkinson’s disease patients in Ethiopia experience sexual dysfunction. Both a reduction in sexual desire and an increase in sexual desire related to levodopa have been reported. Additional crucial findings of this study are a significant and positive correlation between sexual dysfunction with the levodopa equivalent daily dose in male, and with the experience of PD-related stigma, depression, and generalized anxiety in female PD patients. Owing to cultural sensitivity of sexual issues, PD patients may be ashamed to seek help and consult the treating physician. Given the higher prevalence of sexual dysfunction and role of anti-Parkinson’s medication in increasing the burden, it would be advisable that health care providers take a proactive role in initiating sexual health communication. Regular monitoring of medication side effects, and psychosocial intervention can improve sexual health and wellbeing. Given the cross-sectional observational design of the present study, future longitudinal research is needed to examine temporal relationships and potential causal pathways, while interventional studies could further evaluate the effects of medication adjustments and psychosocial interventions on sexual functioning among individuals with PD.

## Figures and Tables

**Table 1:** Socio-demographic characteristics study participants (n = 74).

Variables		Frequency (%)
Age	34–44	17 (23%)
	45–55	57 (77%)
Gender	Male	42 (56.8%)
	Female	32 (43.2%)
Marital status	Single	16 (21.6%)
	Married	29 (39.2%)
	Divorce	18 (24.3%)
	Widowed	7 (9.5%)
	Separated	4 (5.4%)
Educational Status	No formal education	16 (21.6%)
	Primary education	26 (35.1%)
	Secondary education	19 (25.7%)
	Higher education	13 (17.6%)
Religion	Muslim	9 (12.2%)
	Orthodox Christian	50 (67.6%)
	Protestant Christian	15 (20.3)
Residence	Urban	66 (89.2%)
	Rural	8 (10.8%)
Current occupational status	No occupation	38 (51.4%)
	Retired with pension	2 (2.7%)
	Has occupation	34 (45.9)

**Table 2:** Clinical and Psychosocial characteristics of study participants (n = 74).

Variables		Frequency (%)
AO of PD	26–35	4 (5.4%)
	36–45	25 (33.8%)
	46–55	45 (60.8%)
Levodopa daily dose	Not taking levodopa	5 (6.8%)
	125–375 mg	14 (18.9%)
	500–875 mg	42 (56.8%)
	1000–1500 mg	13 (17.6%)
PD stage	Stage 1	8 (10.8)
	Stage 1.5	16 (21.6)
	Stage 2	25 (33.8)
	Stage 2.5	5 (6.8)
	Stage 3	14 (18.9)
	Stage 4	5 (6.8)
	Stage 5	1 (1.4)
Stigma	Mean with StD = 17.65 ± 6.77, Min = 8, max = 35	
Physical activity (in minutes per week)	Median with StD = 97.50 ± 230.86, Min = 0, max = 1260	
Depression	Mean with StD = 9.69 ± 4.76, Min = 0, max = 21	
Generalized anxiety	Mean with StD = 8.49 ± 5.15, Min = 1, max = 21	

Note: AO = age at onset, PD = Parkinson’s disease, StD = standard deviation.

**Table 3:** Correlates of sexual dysfunction in early-onset male PD patients.

	1	2	3	4	5	6	7	8	9
1 Male sexual functioning	1								
2 Age	−0.007	1							
3 Age at the onset PD	0.005	0.767 **	1						
4 Levodopa daily dose	0.411 **	−0.039	−0.117	1					
5 PD stage	0.177	−0.058	−0.298	0.232	1				
6 PD-related stigma	0.279	−0.123	−0.191	0.257	0.373 *	1			
7 Physical activity	−0.066	0.100	0.137	0.016	−0.382 *	0.022	1		
8 Depression	0.104	−0.017	−0.218	0.197	0.483 **	0.344 *	−0.400 **	1	
9 Generalized anxiety	0.123	−0.098	−0.287	0.054	0.499 **	0.394 **	−0.206	0.785 **	1

**. Correlation(r) is significant at the 0.01 level (2-tailed). *. Correlation(r) is significant at the 0.05 level (2-tailed). r = 0.10–0.29 (small), 0.30–0.49 (moderate), ≥0.50 (large).

**Table 4:** Correlates of sexual functioning in early-onset female PD patients.

	1	2	3	4	5	6	7	8	9
1 Female sexual functioning	1								
2 Age	−0.152	1							
3 Age at onset of PD	−0.231	0.858 **	1						
4 Levodopa daily dose	0.109	0.084	−0.070	1					
5 PD stage	−0.035	0.005	−0.172	0.397 *	1				
6 PD-related stigma	0.389 *	−0.280	−0.334	0.057	−0.001	1			
7 Physical activity	−0.212	−0.331	−0.210	−0.206	−0.212	0.119	1		
8 Depression	0.529 **	0.017	−0.096	0.197	0.416 *	0.448 *	−0.245	1	
9 Generalized anxiety	0.629 **	0.018	0.018	0.150	0.097	0.514 **	−0.398 *	0.721 **	1

**. Correlation(r) is significant at the 0.01 level (2-tailed). *. Correlation(r) is significant at the 0.05 level (2-tailed). r = 0.10–0.29 (small), 0.30–0.49 (moderate), ≥0.50 (large).

## Data Availability

The data supporting the findings of this study are available on request from the corresponding author. The data is not publicly available due to privacy or ethical restrictions.

## Abbreviations

AO	Age at onset of PD
ED	Erectile dysfunction
HADS	hospital anxiety and depression scale
HYS	Hoehn and Yahr scale
MIPA	Moderate-intensity physical activity
MOS-SFS	Medical outcomes study sexual functioning scale
PD	Parkinson’s disease
PPSO-E	Parkinson’s patient support organization of Ethiopia
SD	Sexual dysfunction
StD	standard deviation
SSCI-8	stigma scale for chronic illness 8-item version
STROBE	Strengthening the reporting of observational studies in epidemiology
